# *ICE1* of *Pyrus ussuriensis* functions in cold tolerance by enhancing *PuDREBa* transcriptional levels through interacting with PuHHP1

**DOI:** 10.1038/srep17620

**Published:** 2015-12-02

**Authors:** Xiaosan Huang, Kongqing Li, Cong Jin, Shaoling Zhang

**Affiliations:** 1 College of Horticulture, State Key Laboratory of Crop Genetics and Germplasm Enhancement, Nanjing Agricultural University, Nanjing, China, 210095; 2 College of Rural Development, Nanjing Agricultural University, Nanjing, China, 210095

## Abstract

ICE1 transcription factor plays an important role in plant cold stress via regulating the expression of stress-responsive genes. In this study, a *PuICE1* gene isolated from *Pyrus ussuriensis* was characterized for its function in cold tolerance. The expression levels of the *PuICE1* were induced by cold, dehydration and salt, with the greatest induction under cold conditions. PuICE1 was localized in the nucleus and could bind specifically to the MYC element in the *PuDREBa* promoter. The PuICE1 fused to the GAL4 DNA-binding domain to have transcriptional activation activity. Ectopic expression of the *PuICE1* in tomato conferred enhanced tolerance to cold stress at cold temperatures, less electrolyte leakage, less MDA content, higher chlorophyll content, higher survival rate, higher proline content, higher activities of enzymes. In additon, steady-state mRNA levels of six stress-responsive genes coding for either functional or regulatory genes were induced to higher levels in the transgenic lines by cold stress. Yeast two-hybrid, transient assay, split luciferase complementation and BiFC assays all revealed that PuHHP1 protein can physically interact with PuICE1. Taken together, these results demonstrated that *PuICE1* plays a positive role in cold tolerance, which may be due to enhancement of *PuDREBa* transcriptional levels through interacting with the PuHHP1.

Cold is one of the most devastating abiotic stresses that impair plant growth and development, reduce productivity, and limit geographical distribution of natural populations. Therefore, enhancement of cold tolerance has been a major subject of considerable research interest over a long period. Although some manual measures can be used to enhance cold tolerance, the last aim is to create tolerant cultivars. As a supplementation for traditional breeding, biotechnology-mediated approach has been proven to be an effective approach for generating novel germplasms with elevated stress tolerance. A vast number of elegant studies have provided evidence showing that genetic engineering is a powerful strategy for creating germplasms with enhanced cold tolerance^1^,^2^.

As sessile organisms, plants have developed arrays of molecular, physiological and biochemical strategies to adapt to the adverse conditions^3^,^4^,^5^,^6^,^7^. Over the last decades, enormous progress has been made in deciphering significant components implicated in the cold signalling network^8^. As a result, our understanding on the cold response has been greatly accelerated. It is now accepted that one of the most important milestones has been the identification of C-repeat-binding factor (CBF) genes, including CBF1, CBF2, and CBF3^9^,^10^,^11^. Another important breakthrough has been characterization of Inducer of CBF Expression in *Arabidopsis thaliana* (*AtICE1*), an upstream transcription factor that regulates the transcription of CBF genes in the cold.

ICE1 encodes a MYC-like bHLH transcriptional activator, which could bind specifically to the MYC recognition sequences in the CBF promoter and activate CBF transcription^12^. Overexpression of ICE1 in wild-type plants enhanced the expression of *CBF3* in *A. thaliana*, resulting in an increased transcription level of the downstream *COR* genes, such as *COR15* and *COR47*, thus increasing the chilling and freezing tolerance of the plants, but induction of these genes were impaired in an ice1 mutant. Further in-depth work showed that ICE1 could regulate the expression of CBF3 by binding specifically to MYC-recognizing (MYCR) core sequence (CANNTG) in the CBF3 promoter region^12^,^13^. In addition, ICE2, an ICE1 homologue in *Arabidopsis thaliana*, was identified as functioning in regulating cold tolerance via modulation of CBF1 expression^14^. These findings suggest that ICE1 or ICE2 acts as a master regulator of the cold signalling pathway and plays a pivotal role in mediating plant responses to cold, thus establishing ICE1–CBF–COR as the most significant signalling cascade. To date, ICE1 and ICE2 homologues have been identified in *Arabidopsis*, various plants, such as Calellia sinensis^15^, apple^16^, tomato^17^, Chrysanthemum dichrum^18^, trifoliate orange^19^, Phalaenopsis aphrodite^20^, and banana^21^. In additon, All of these successful examples indicate that ICE1 functions in cold tolerance due to its essential role in governing the expression of CBFs, which in turn regulate the downstream target genes^4^,^12^.

Although ICE1 members have been comprehensively studied in the model plants, information concerning their counterparts in woody plants is relatively lacking. Pear is the one of the most widespread fruit in the word, with great economic and health value. There exist many wild relatives of cultivated pear (*Pyrus ussuriensis*) with different degrees of tolerance to abiotic stress^22^. *Pyrus ussuriensis*, an important rootstock for pear, is cold tolerant, making it a good source of valuable genes involved in cold stress tolerance. A raised question is whether or not the ICE1 homologue in *Pyrus ussuriensis* can function in stress tolerance. In addition, it was noticed that limited information is available concerning before physiological and/or molecular basis of enhanced stress tolerance in the transgenic plants overexpressing an ICE1 gene in previous reports. Therefore, we tried to clone an ICE1 gene from *Pyrus ussuriensis*, and functionally characterize its role in cold tolerance. In addition, higher activities of enzymes and higher expression of stress-responsive genes were identified in transgenic plants. Yeast two-hybrid, transient assay, split luciferase complementation and BiFC assays all revealed that the PuHHP1 protein were physically associated with PuICE1 and stimulated *PuDREBa* transcriptional activities. Taken together, our findings suggest that *PuICE1* is involved in the regulation of cold tolerance via modulating *PuDREBa* transcriptional levels through interacting with the *PuHHP1* gene.

## Results

### Cloning and sequence analysis of *PuICE1*

**Figure 1:**
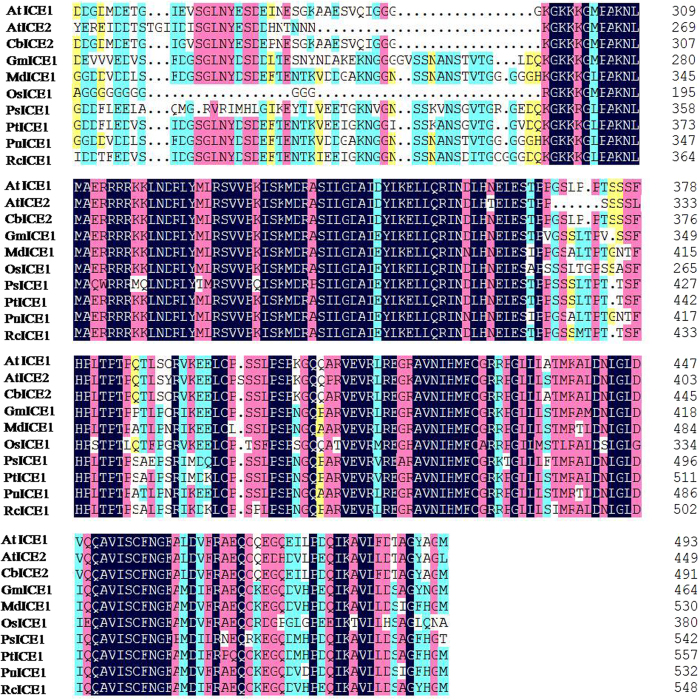
Multiple alignments of the deduced amino acid sequence of PuICE1 and those of *Populus trichocarpa* (PtICE1, ABN58427), *Populus suaveolens* (PsICE1, ABF48720), *Ricinus communis* (RcICE1, EEF51703), soybean (GmICE1, ACJ39211), *Arabidopsis thaliana* (AtICE1, AAP14668; AtICE2, BAC42644), *Capsella bursa-pastoris* (CbICE1, AAS79350), *Malus domestica* (MdICE1, ABS50251), *Oryza sativa* (OsICE1, NP_001045272). Identical and similar residues are shown in black and gray background, respectively. The multiple alignment was performed with ClustalW2 using the default parameters.

More and more reports demonstrate that plant ICE1-like genes play an important role in cold tolerance, but ICE1 genes have not yet been characterized from *Pyrus ussuriensis*. To confirm whether *PuICE1* gene from *Pyrus ussuriensis* also functions in cold tolerance, we searched the Pear Genome database using the *AtICE1* sequence as bait, which yielded seven outputs. The first output with high identity to the *AtICE1* gene was selected to further analyse in this study. RT-PCR amplification of the cDNA using the primers designed from the conting yielded a single fragment, which was verified as 2602 bp by sequencing. The sequence showed a high degree of homology to *AtICE1* genes in the database, indicating that it was an ICE1 gene homologue. Bioinformatics analysis showed that the cDNA, 2602 bp in length, contained a 1602-bp open reading frame (ORF), along with a 500-bp 5′ untranslated region (UTR) and a 500-bp 3′ UTR. The cDNA, designated as *PuICE1* (*Pyrus ussuriensis* ICE1), encodes a predicted polypeptide of 533 amino acids with a calculated molecular weight of 57 kDa and a *pI* of 5.52. Motif scanning against MyHits (http://myhits.isb-sib.ch/cgi-bin/motif_scan) showed that the amino acids of the PuICE1 contains 48 amino acids composed of an typical bHLH domain (positions 344–391) which be composed of a 15 amino acids basic region and two helices (14 amino acid each) that were connected by a loop of five amino acids (Fig. 1). Multiple alignments between PuICE1 and 9 other plant ICE1 proteins shared high sequence identity among each other, PuICE1 had 80% sequence identity to MdICE1 of apple (Malus domestica) and 59% to ICE1 of *Arabidopsis* (Fig. 1).

### Expression pattern of *PuICE1* under various stress treatments

**Figure 2:**
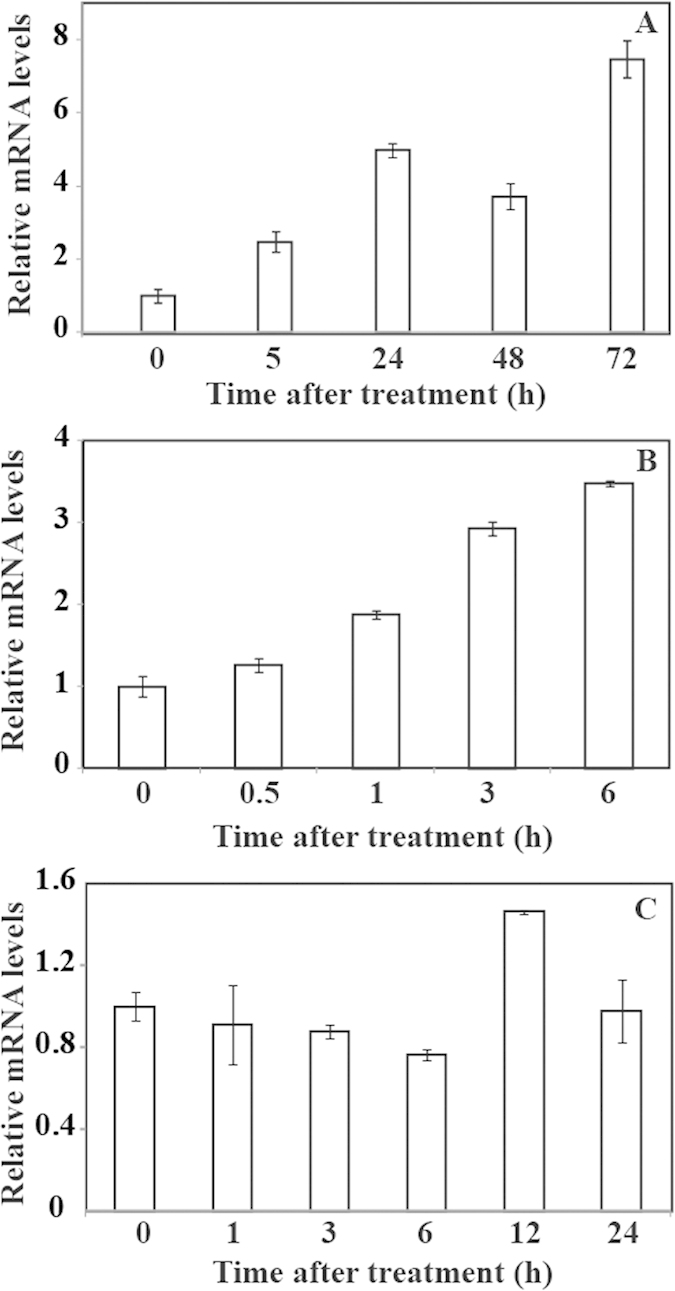
Time-course expression levels of *PuICE1* in *Pyrus ussuriensis* under abiotic stresses. (**A**–**C**) expression patterns of *PuICE1* in response to cold (**A**), dehydration (**B**) and salt stress (**C**). The samples were collected at the designated time points and analyzed by qPCR. Error bars stand for SD based on four replicates.

In order to investigate the response of *PuICE1* to abiotic stress, real-time quantitative PCR (qPCR) was used to examine the expression pattern of *PuICE1* by qPCR. Under cold treatment, the transcript level of *PuICE1* was pronouncedly induced by nearly 3 fold, and then increased progressively to its highest level at 72 h (more than 7-fold the initial level) (Fig. 2A). The transcript level of *PuICE1* began to accumulate 0.5 h after dehydration, and continued to increase until it reached to the highest level at 6 h (Fig. 2B). Upon exposure to salt stress, the transcript level of *PuICE1* did not change notably except a slight decrease at 12 h (Fig. 2C), indicating that *PuICE1* was not clearly salt-inducible.

### PuICE1 was localized in the nucleus and PuHHP1 was localized in the plasma membrane

**Figure 3:**
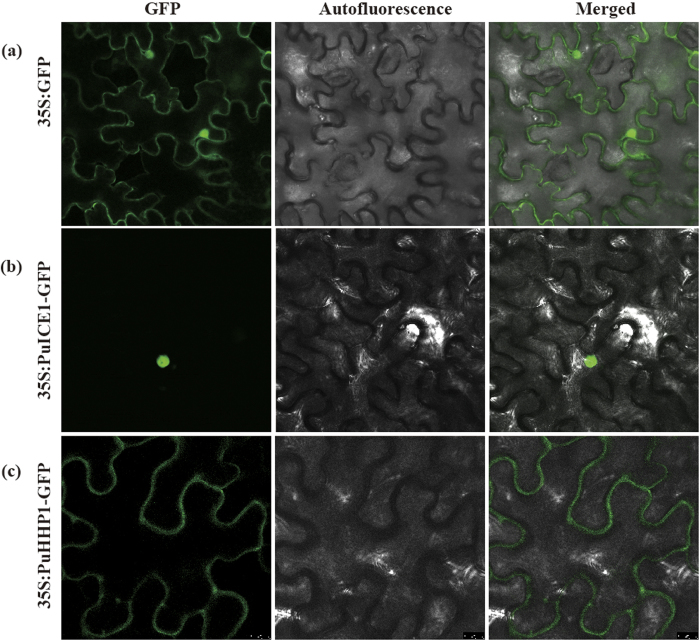
Subcellular localization of PuICE1 and PuHHP1. Tobacco epidermal cells were transiently transformed with constructs containing either control (GFP alone, (**a**)) or fusion plasmid (PuICE1: GFP, (**b**) PuHHP1: GFP, (**c**)). Images under blight field (middle), fluorescence (left) and the merged images are shown on the right.

Sequence analysis showed that there was one nuclear localization signal (position, 339–356), implying that it may be localized in the nucleus. To confirm this, the *PuICE1* coding region was fused to the N–terminus of the GFP reporter gene under the control of the cauliflower mosaic virus 35S promoter. The localization of the fusion protein (*PuICE1*) and the control (GFP) was analyzed in tobacco leaf epidermis via *Agrobacterium*–mediated transformation. Microscopic visualization showed that the control GFP was uniformly distributed throughout the whole cell (Fig. 3a), whereas the PuICE1–GFP fusion protein was observed exclusively in the nucleus (Fig. 3b). These results indicated that PuICE1 was a nuclear protein.

To find out the subcellular localization of PuHHP1 before and after cold, full length ORF of PuHHP1 was fused to N–terminal of GFP reporter protein driven by CaMV 35S promoter, generating a fusion protein PuHHP1: GFP. The fusion protein were separately infiltrated into tobacco epidermal cells. Microscopic observation demonstrated that green fluorescence was distributed in plasma membrane under normal condition (Fig. 3c), whereas green fluorescence was also detected in the plasma membrane (data not shown) when the infected tobacco were exposed to cold stress (4 °C for 12 h), indicating that PuHHP1 was a membrane protein, which location was not affected by cold stress.

### PuICE1 activates gene expression and binds to the MYC element in the *PuDREBa* promoter

**Figure 4:**
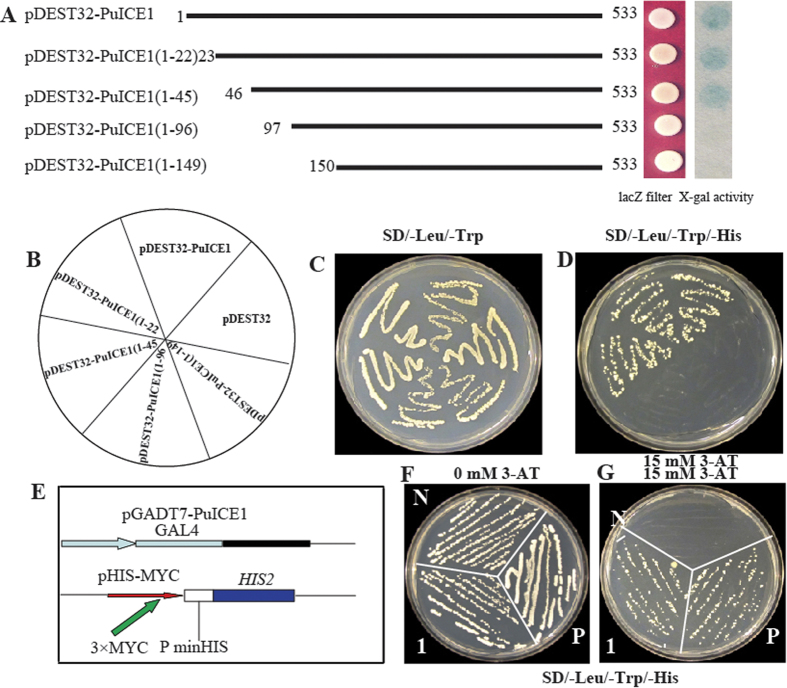
Transcriptional activation assay of PuICE1 in yeast. (**A**–**D**) Transactivation assay of completed or truncated PuICE1. (**A**) Schematic diagrams indicating full length (FL) or deleted PuICE1 (I1-I5); X–gal assay of the transformed yeast cells grown on SD/–Leu/–Trp/–His added with 15 mM 3–AT and 20 mM X-α-gal. (**C**,**D**) Growth of the yeast cells transformed with different plasmids on SD/–His/–Leu/–Trp added with 15 mM 3-AT. (**E**–**G**) Schematic illustration of the vectors (pGADT7–PuICE1, pHIS2–MYC) used for transactivation assay. (**F**,**G**) Growth of the yeast cells co–transformed with vectors of positive control (P), negative control (N) and pGADT7–PuICE1 with pHIS2–MYC (1) on SD/–Leu/–Trp/–His added with 0 or 15 mM 3–AT.

The transactivation activity is another important feature for a transcription factor. To determine which region of PuICE1 acts as transcriptional transactivation, yeast two-hybrid assays were carried out using intact or truncated PuICE1 as an effector (Fig. 4A–D). The transfected yeast cells harboring either the full-length PuICE1 (pDEST32–PuICE1) or the truncated version (pDEST32–PuICE1(1–22a); pDEST32–PuICE1(1–46a)) grew well on the selection medium, suggesting that the N-terminal 45 residues are dispensable for transactivation activity of the PuICE1. On the contrary, when the pDEST32–PuICE1 (1–96 aa); pDEST32–PuICE1 (1–149 aa) of amino acids at the N–terminal were deleted, no interaction was detected, which was further supported by the colony–lift filter assay (Fig. 4A–D). Taken together, these results demonstrate that amino acids from positions 46 to 95 in PuICE1 are critical for the transactivation activity of PuICE1.

ICE1 of *Arabidopsis thaliana* can bind to the cis–element MYCR in the promoter of gene AtCBF3^12^, which compelled us to identify whether PuICE1 could also bind to a sequence containing the MYC recognition sites in the *PuDREBa* promoter, the open reading frame of *PuICE1* gene was fused to the GAL4 activation domain of the pGADT7 and the fused construct (pGADT7–PuICE1) was co–tansformed with pHIS–MYC containing triple tandem repeats of the MYC were co–transformed into yeast strain Y187. The results showed that only the combination of pGADT7 and pHIS–MYCR grew normally on the SD/–Leu/–Trp/–His medium supplied with 15 mM 3–AT (Fig. 4E–G), indicating that PuICE1 could bind to the MYC recognition sites in the *PuDREBa* promoter and activates the HIS reporter gene in yeast.

### Overexpression of *PuICE1* increases the cold tolerance of transgenic plants

To investigate the function of *PuICE1*, *Agrobacterium*-mediated transformation of tomato leaf discs was carried out using a binary vector containing *PuICE1* under the control of 35S promoter of cauliflower mosaic virus (CaMV 35S). Totally, 6 T0 lines were characterized by PCR with primers specific to *PuICE1* (GSP1, Table S1), and 4 out of them were confirmed as putative transgenic lines, and overexpression of *PuICE1* in two lines (TG8 and TG10) was verified by semi-quantitative RT-PCR analysis (Fig. S1).

**Figure 5:**
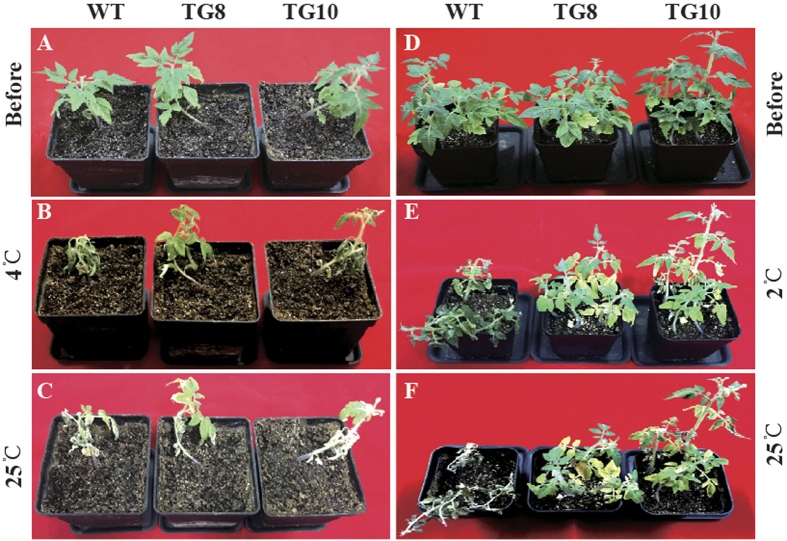
Overexpression of *PuICE1* conferred enhanced cold tolerance in tomato. (**A**–**C**) Plant phenotype of tomato wild type (WT) and transgenic plants (TG8 and TG10) before and after cold treatment for 3 d at 4 °C, followed by recovery growth for 5 d at ambient environment. (**D**–**F**) Plant phenotype of tomato wild type (WT) and transgenic plants (TG8 and TG10) before and after cold treatment for 3 d at 2 °C, followed by recovery growth for 5 d at ambient environment.

**Figure 6:**
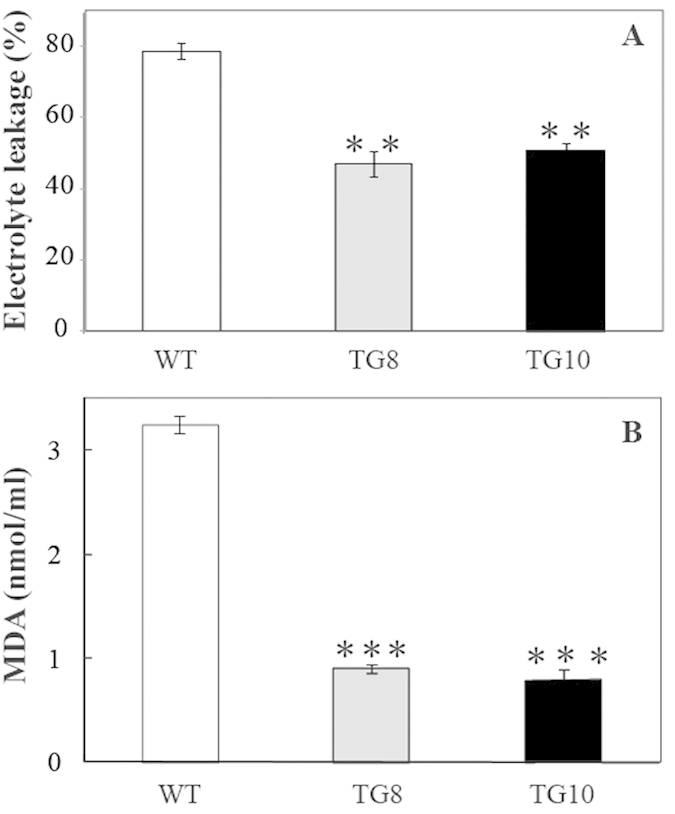
Measurement of MDA (**A**) and electrolyte leakage (**B**) in the overexpressing lines (TG8 and TG10) and the controls (WT) after chilling treatment for 3 d at 2 °C.

**Figure 7:**
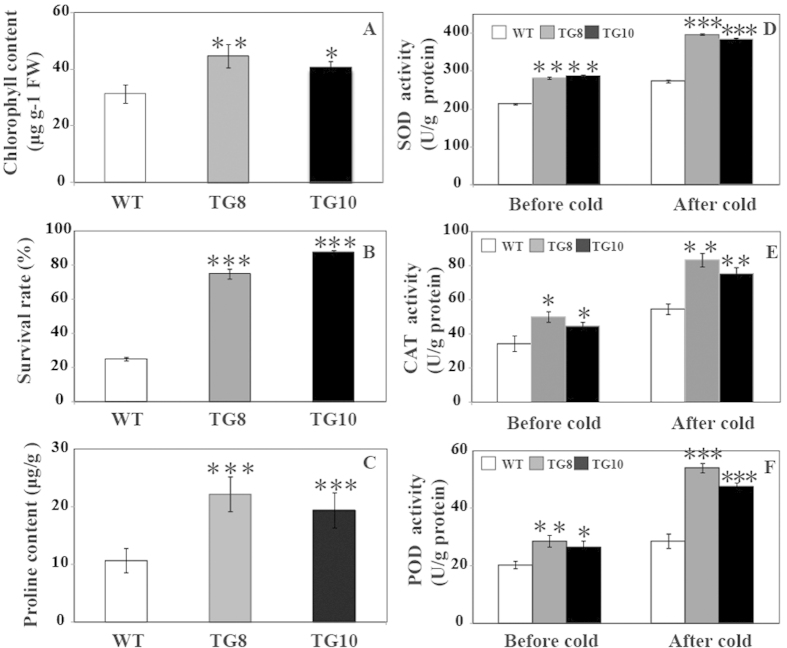
Overexpression of *PuICE1* conferred enhanced cold tolerance in tomato. (**A**) Plant phenotypes of wild type (WT) and transgenic lines (TG8 and TG10) before and after chilling treatment (2 °C for 3 d). (**A**) Chlorophyll contents (**B**) Survival rate (**C**) Proline content (**D**–**F**) Activity of SOD, CAT and POD in tomato WT and transgenic lines, analyzed before and after chilling treatment.

To evaluate the function of *PuICE1* in cold tolerance, T2 transgenic tomato plants and the WT were subjected to cold treatment at 4 °C or 2 °C. Under the normal growth conditions, no difference in morphology was showed between the transgenic and the wild-type. When cold treated at 4 °C for 4 d using 30-d-old seedlings, chilling injury was observed in the leaves of the wild-type plants, but the transgenic plants were not affected (Fig. 5B). After recovery at room temperature for 5 d, most of WT died, while the two transgenic lines grew well (Fig. 5C). When the plants were exposed to cold stress (2 °C for 3 d), the transgenic lines displayed less serious damage in comparison with the WT (Fig. 5E). Electrolyte leakage, a reliable indicator of cell membrane damage caused by abiotic stresses, was used to indicate the stress tolerance capacity. At the end of cold stress, electrolyte leakage of TG8 (46%) and TG10 (50.0%) plants was significantly lower than that of the WT (78.0%) (Fig. 6A). In addition, the MDA level exhibited a profile similar to the EL, significantly lower in the transgenic lines relative to the WT (Fig. 6B). After the chilling treatment, the total chlorophyll of the transgenic lines (44.66 μg g-1 FW for TG8 and 40.66 μg g-1 FW for TG10) was significantly higher than WT (31.33 μg g-1 FW, Fig. 7A). After recovery growth for 5 d in an ambient environment, the survival rate of WT plants was 25%, significantly lower than that of the transgenic lines: 75% for TG8 and 87.5% for TG10 (Fig. 7B).

The activity of three significant antioxidant enzymes (SOD, CAT, and POD) and the level of several important metabolites were assessed in the leaves sampled from the potted plants before and after cold treatment. under normal growth conditions, activities of the three enzymes were higher than those of the control, but the difference was prominent. Cold stress caused increase of SOD activity, which was significantly lower in WT than in TG8 and TG10 (Fig. 7D). Activity of CAT was augmented in all of the tested samples, while the transgenic lines had significantly higher activities than WT (Fig. 7E). Exposure to cold resulted in slight rise of POD activity in WT, which was notably enhanced in the two transgenic lines. As a result, POD activity of TG8 and TG10 was 1.9 and 1.7 folds of that in WT, respectively (Fig. 7F). All of these showed that activities of the three detoxifying enzyme were significantly higher in the transgenic lines than WT.

### Expression analysis of stress-responsive genes before and after cold treatment

**Figure 8:**
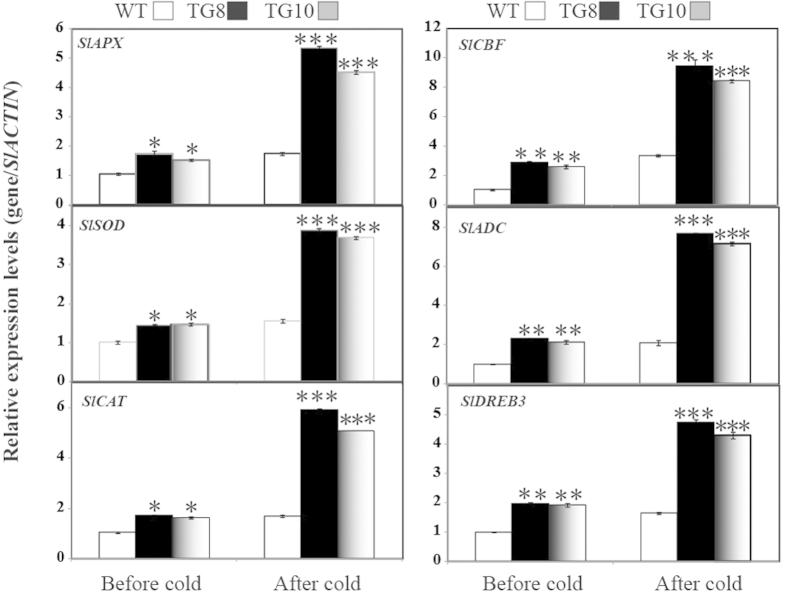
Expression profiles of the six stress–responsive genes in wild type (WT), transgenic lines (TG8 and TG10) before and after cold treatment. RNA was extracted from leaves sampled at the onset and before after 3 d of cold stress, and reverse transcribed to synthesize cDNA, which was used for RT–PCR analysiswith primers specific for these genes. mRNA levels of these geneswere normalized to the transcripts of *Actin* in the same samples.

To gain further insight into the molecular mechanism underlying the enhanced cold resistance in the transgenic plants, the transcript abundance of 6 ROS–related or stress–responsive genes was examined in the WT and transgenic plants before and after 3 d cold treatment at 2 °C (Fig. 8). These genes encode enzymes for direct ROS detoxification (*SlAPX*, *SlCAT* and *SlSOD*), enzymes involved in biosynthesis of polyamine (*SlADC2*), and significant regulatory protein (*SlCBF* and *SlDREB3*). Under normal conditions, mRNA levels of all 6 genes in TG8 and TG10 were higher than those in the WT. Exposure to cold treatment caused up-regulation of the transcript levels of the analysed genes in two lines, but TG8 and TG10 still had a significantly higher expression level in comparison with the WT. These data indicated that overexpression of *PuICE1* in tomato enhances the transcript levels of the ROS-related and stress-related genes with or without cold stress.

### PuICE1 could interact with PuHHP1

**Figure 9:**
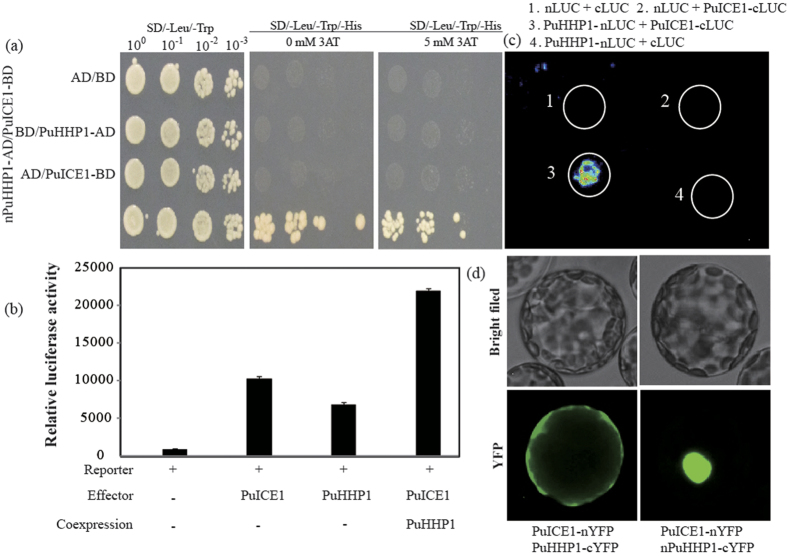
Protein–Protein interaction between PuICE1 and PuHHP1 verified by Y2H (**a**), transient assay (**b**), split luciferase complementation assay (**c**), or BiFC (**d**). (**a**) Growth of yeast cells of negative control and co–transformants of PuICE1 and PuHHP1 on SD/–Leu/–Trp or SD/–Leu/–Trp/–His added with or without 5 mM 3–AT. (**b**) Transcriptional regulation of *PuDREBa* by the PuICE1–PuHHP1, Effector and reporter reconstitution were co–transformed into Arabidopsis protoplasts. Three independent measurements of Relative luciferase activities were averaged. Bars indicate the standard error of the mean. (**c**) the PuICE1 interactions with PuHHP1 was also confirmed using a split luciferase complementation assay in tobacco leaves. (**d**) Protein–protein interaction between PuICE1 and PuHHP1 verified by BiFC. PuICE1–nYFP and PuHHP1–cYFP (left), PuICE1–nYFP and nPuHHP1–cYFP (right).

Molecular mechanisms of the gene *PuICE1*-mediated regulation of the *Pyrus ussuriensis* in cold tolerance was further elucidated, previous report showed that HHP1 could interact with ICE1 to regulate its transcriptional activity in *Arabidopsis*^23^,^24^. In order to investigate whether PuHHP1 can also interact with PuICE1 in *Pyrus ussuriensis*, the N-terminal fragment of PuHHP1 (1–108 aa) was fused to the GAL4 activation domain of vector pDEST22 and the fused construct (pDEST22-PuHHP1) was co-transformed with pDEST32-PuICE1 construct into yeast strain MaV203. As shown in Fig. 9a, the yeast cells could grow on the yeast cells could grow on SD/–Leu/–Trp/–His medium. When 5 mM 3–AT was added to the medium, only yeast cells of co–transformant with PuICE1 and PuHHP1 grew normally. Y2H assay indicated that PuICE1 could interact with PuHHP1.

Given that PuHHP1 interactes with PuICE1, it is highly probable that PuHHP1 may also be involved in the control of *PuDREBa* expression. To identify this hypothesis, the transient expression analysis using *Arabidopsis* protoplasts was carried out. As expected, transfection of *PuICE1* alone in *Arabidopsis* protoplasts induced a high level of *PuDREBa*–LUC expression, in agreement with above results. Interestingly, transfection of *PuHHP1* alone in arabidopsis protoplasts also induced a high level of *PuDREBa*–LUC expression. Compared with transfection of *PuHHP1* alone or *PuICE1* alone, transfection of *PuICE1* together with *PuHHP1* resulted in a signicant higher levels of *PuDREBa*–LUC expression (Fig. 9b). The results indicate the PuHHP1 and PuICE1 are mutually interconnected in transcriptional regulation of *PuDREBa*.

The PuICE1 interactions with PuICE1 was also confirmed using a split luciferase complementation assay in tobacco leaves. Interestingly, the relative stronger luciferase activities was observed in tobacco epidermis transformed with vector containing PuICE1–nLUC and PuHHP1–cLUC. In contrast, there was no luciferase activities in the cells transformed with vectors of the negtive control (Fig. 9c). Y2H experiments showed that protein-protein interaction occurred between PuICE1 and the N–terminal (1–108 aa) of PuHHP1. A BiFC assay was further performed to verify this interaction in planta (Fig. 9d). To examine the co–localization of full length PuHHP1 and PuICE1 before and after cold treatment, PuICE1–nYFP and PuHHP1–cYFP were co-transformed into *Arabidopsis* mesophyll protoplasts by PEG transformation. Co-expression of PuICE1–nYFP and PuHHP1–cYFP under normal condition (Fig. 9d), YFP fluorescence was detected predominantly in the plasma membrane in *Arabidopsis* protoplast, indicating that PuICE1 can interact with PuHHP1 in the plasma membrane. However, co-expression of PuICE1–nYFP and PuHHP1–cYFP under cold stress (4 °C for 12 h), YFP fluorescence was also detected predominantly in the plasma membrane (data not shown), indicating that co–localization of PuICE1–PuHHP1 was also not affected by cold stress, which is consistent with the location of PuHHP1 was not affected by cold. To futher determine which region of PuHHP1 interacted with PuICE1, *Arabidopsis* mesophyll protoplasts were co–transformed with nPuHHP1–cYFP, coding for the N-terminal 108 aa of PuHHP1, plus PuICE1–nYFP. Interestingly, green fluorescence was inside the nucleus when co–expresssing nPuHHP1 and PuICE1 (Fig. 9d), indicating co–expression of PuICE1 and nPuHHP1 may move into a specific site in the nucleus. Taken together, Y2H, Transient expression analysis, split luciferase complementation and BiFC assays all indicated that PuICE1 could interact with PuHHP1, and the N–terminal of PuHHP1 (1–108 aa) was responsible for its interaction with PuICE1 (Fig. 9).

## Discussion

Plants have a wide range of TFs; for instance, the Arabidopsis (Arabidopsis thaliana) genome contains more than 1,500 TFs, accounting for nearly 6% of its total genes^25^. Among the TFs, the basic helix-loop-helix (bHLH) motif-containing TFs are important regulatory components of the transcriptional networks. 177 and 167 bHLHs have been unravelled in the genomes of rice^26^ and *Arabidopsis thaliana*^27^. To date, plant bHLH proteins have been shown to function in the transcriptional regulation of a diversity of biological processes, including flowering^28^, trichome or root hair development^29^,^30^,^31^, chloroplast development^32^, biosynthesis of flavonoid, isoquinoline alkaloid, and anthocyanin^33^,^34^,^35^,^36^, and nodule vascular patterning^37^. Furthermore, some plant bHLH TFs are responsive to abiotic stresses. For example, INDUCER OF CBF EXPRESSION1 (ICE1) and ICE2 of Arabidopsis and MdCIbHLH1 of apple (Malus domestica) were suggested to be involved in the cold stress response^12^,^14^,^16^. OsbHLH148, a rice(Oryza sativa) bHLH gene, functioned in drought tolerance as a component of the jasmonate signaling module^38^. ICE1 is a well-characterized bHLH protein that acts as an upstream regulator of the transcriptional regulation cascade of the cold response in *Arabidopsis*^27^. However, little is known about the roles of ICE1 homologues in *Pyrus ussuriensis*, a very cold-hardy plant. Thus, characterization of an ICE1 gene of *Pyrus ussuriensis* is crucial to decipher the cold signalling pathway pertinent to freezing tolerance and to provide valuable gene candidates for genetic manipulation.

Here, we report the identification of a MYC-like bHLH transcription factor (*PuICE1*) in *Pyrus ussuriensis*. Multiple sequence alignment suggests that the bHLH domain and zipper region of PuICE1 share striking sequence similarities with those of the bHLH proteins from other plants, despite a low degree of sequence conservation outside the bHLH domain. According to the previous report, PuICE1 should be classified into the category of E-box binders as it contains two specific residues, glutamate (E) and arginine (R), in the basic region^27^. These observations seem to suggest that PuICE1 might be a novel putative ICE1 homologue of *Pyrus ussuriensis*.

An important feature of plant bHLHs is the induction of their transcript levels by abiotic stresses^19^,^36^. qRT-PCR analysis demonstrated that steady state mRNA levels of PuICE1 were induced by cold and low temperature. Expression patterns of *PuICE1* were largely similar to *AtICE1* that has been shown to be induced by salt, cold and drought (Fig. 2). However, it has to be mentioned that *PuICE1* was not induced by salt, different from *AtICE1*. The disparity of expression patterns between *PuICE1* and *AtICE1* in response to dehydration might be presumably ascribed to the inherent difference in plant species. However, durations of dehydration treatment in these studies may also account for the difference, as shorter time frame (30 min) was used for *Arabidopsis thaliana* in comparison with our work. Interestingly, from the Fig. 2 we can see, PuICE1 transcript level increased progressively under cold stress until reaching the highest level at 72 h (greater than 8-fold induction), which is not consistent with previous results that ICE1 was expressed constitutively, the expression levels of ICE1were stable duing different low temperature treatments^12^. The strongest induction of *PuICE1* transcript by cold stress forced us to elucidate its function in cold tolerance. The assays demonstrated that overexpression of *PuICE1* in tomato resulted in pronouncedly enhanced tolerance to cold stresses, indicating that PuICE1 acts as a positive regulator of cold signalling cascade. Meanwhile, overexpression of *PuICE1* did not cause negative impacts on plant growth of the transgenic lines under normal growth conditions, suggesting that *PuICE1* might hold great potential for genetic engineering to improve cold tolerance.

Compared with dehydration and salt, low temperature caused more profound induction of *PuICE1* mRNA abundance, which compelled us to do in-depth work on elucidation of the potential role of this gene for enhancing cold tolerance by generating transgenic plants transformed with overexpression. To this end, *PuICE1* was transformed into tomato, a model plant that has been extensively used for functional analysis of genes from many plants. Overexpression of *PuICE1* in tomato pronouncedly conferred enhanced tolerance to cold stress under chilling temperature, as measured by electrolyte leakage, survival rate, and chlorophyll content, along with phenotypic observation. These data demonstrate that *PuICE1* plays a positive regulatory role in cold tolerance. Our work agreed with earlier reports, in which overexpression of bHLH family members has been shown to render tolerance to multiple stresses in the same transgenic lines^18^,^19^,^36^,^39^, implying that bHLHs class transcription factors hold great potential for genetically manipulating stress tolerance.

Despite the fact transformation of *PuICE1* genes led to improvement of abiotic stress tolerance, the mechanism underling the tolerance remained largely unknown. This stimulated us to carry out more work to find out physiological and molecular difference between the transgenic plants and WT under cold stress. It was found that TG8 and TG10 contained higher levels of antioxidants such as higher activities of SOD, POD, and CAT in comparison with the WT before cold stress. This indicates that over-expression of the *PuICE1* gene has facilitated the activation of the antioxidant defence system even in the absence of stresses. This provides convincing evidence to show that the *PuICE1* functions in cold tolerance by, at least partially, the activation of the enzyme activities.

To cope with unfavorable environmental constraints plants modulate the expression of a large spectrum of stress-responsive genes, constituting an important molecular basis for the response and adaptation of plants to stresses^40^,^41^,^42^. In order to understand regulatory function of *PuICE1* and to explain the enhanced cold tolerance at molecular levels, transcript levels of 6 stress-responsive genes (*SlAPX*, *SlSOD*, *SlCAT*, *SlCBF*, *SlADC*, *SlDREB3*) were monitored before and after cold treatment, these genes in other plants have been shown to be involved in abiotic stress response^18^,^43^,^44^,^45^,^46^. qRT-PCR analysis showed that steady-state mRNA levels of these genes were higher in the transgenic plants compared with those of WT in the absence of cold stress, in line with earlier reports in which overexpression of a TF resulted in extensive alteration of transcript levels of an arsenal of related genes^47^,^48^. Although expression levels of all of the tested genes were upregulated by cold, they were still higher in the transgenic plants than in WT, indicating that these genes were more intensely induced in the transgenic lines. It was found that transcript levels of the genes encoding ROS-scavenging enzymes (*SlAPX*, *SlSOD*, *SlCAT*) were up-regulated in the *PuICE1*-overexpressing lines under normal or cold treatment, consistent with the greater activity of these antioxidant enzymes. This may presumably explain the activation of the antioxidant enzymes in the transgenic lines. On the other hand, one gene (*SlADC*) involved in polyamine synthesis were also induced to a higher level in the transgenic lines relative to the WT. Polyamines are important stress molecules that play critical roles in abiotic stress tolerance due to chemical and physical interactions with macromole cules including nucleic acids, phospholipids, and proteins^48^. More drastic induction of these genes implied that the transgenic plants might synthesize higher levels of polyamines to prevent them from lethal injury and maintain better growth under cold stress. Interestingly, the expression patterns of *SlCBF* and *SlDREB3* were enhanced in TG8 and TG10 as compared with the WT before and after cold, which is consistent with the result obtained by Chinnusamy *et al.* (2003) who showed that cold-induced modification of the AtICE1 protein or of a transcriptional cofactor may be necessary for AtICE1 to activate the expression of CBFs. These results suggest that PuICE1 acts as a signal transduction component in the CBF pathway and is associated with cold tolerance, similar to *ICE1* genes in Arabidopsis and wheat^12^,^49^. In the future, extra work is needed to decipher the connection between these genes so as to gain more insight into the molecular mechanisms underlying *PuICE1* function in cold stress tolerance.

During the last decades our understanding on plant cold response has been greatly advanced^50^. The signal transduction networks on cold response are becoming increasingly clear, *ICE1* plays a critical role in cold response by positively regulating *CBF3* through binding specifically to the MYCR element in the promoter region^12^. This regulation is considered as a classical mode of action on *ICE1*, which is also reasonable as *ICE1*, encoding a bHLH transcription factor, might function in cold signalling via transcriptional regulation of its target genes. However, it is worth mentioning that, as protein-protein interactions are important for executing gene function, exploration of PuICE1-interacting protein may shed new light on the mechanisms underlying enhanced cold tolerance from a different aspect. As a matter of fact, bHLH proteins have been revealed to interact with other non-bHLH transcription factors or functional proteins, forming protein complexes, to participate in various cellular processes. For example, the MYB15 protein interacts with ICE1 and binds to Myb recognition sequences in the promoters of CBF genes^51^. JAZ1 and JAZ4 interact with and repress the transcriptional function of ICE1 in Arabidopsis^52^. ABF-ICE1 interaction regulates stomatal development^53^. OST1 kinase modulates freezing tolerance by enhancing ICE1 stability in *Arabidopsis*^54^. In another work, a HEPTAHELICAL PROTEIN 1 (HHP1) protein of Arabidopsis, HHP1 is transcriptionaly induced by cold, and activated HHP1 protein interacts with the MYC-type basic helix-loop-helix (bHLH) trancription factor ICE1^23^,^55^. In this study, the PuICE1 protein was found to bind to the MYC recognition site of the *PuDREBa* promoter.

Yeast two–hybrid and Split Luciferase complementation Assays revealed that PuHHP1 protein can interact with PuICE1, Transient expression analysis indicate the PuHHP1 interact specifically with *PuDREBa* upstream regulators (PuICE1) to strengthen their transcriptional activity probably by triggering post-translational modifications and individually regulate *PuDREBa* expression in response to cold stress. These results agree with earlier reports that the ICE1 protein interacting with HHP1, participate in the ABA-independent signalling pathway in response to cold^41^. The localization of PuHHP1 is in the plasma membrane under noramal or cold conditions, a question is thus raised as to the localization of PuHHP1 in the plasma membrane might not be compatible with the function of ICE1. It is now clear that post-transcriptional modification, such as sumoylation and ubiquitination^56^,^57^, influence the regulation of ICE1 on CBF3 under cold conditions. However, it remains to be investigated whether affection the co-localization of PuICE1-PuHHP1 before and after cold treatment in *Arabidopsis* protoplasts. BiFC result showed that co-expression of PuICE1–nYFP and PuHHP1–cYFP, YFP fluorescence was detected prodoplast in plasma membrane under normal condition. Surprisingly, co–localization of PuICE1–PuHHP1 was also detected in the plasma membrane in transformmed protoplasts under cold stress. Indicating co-localization of PuICE1 and PuHHP1 was also not affected by cold stress. Interestingly, co-expressing PuICE1 and nPuHHP1 (N–terminal domain, 1–108 aa) was inside the nucleus, indicating that these two protein complex may enter into a specific site in the nucleus. One possibility of the existence of other unexplored mechanisms is that PuICE1 dissociates from PuHHP1 when necessary, or, alternatively, it should be mentioned that there might be the N–terminal of PuHHP1 may be released by controlled proteolysis. In the future, more work is required to experimentally clarify physiological mechanism between PuHHP1 and PuICE1, and to decipher their role in cold tolerance.

Taken together, PuICE1 of *Pyrus ussuriensis* was upregulated by various abiotic stresses, such as cold and dehydration, as it was induced by cold stress in a stronger manner, transgenic tomato plants overexpressing PuICE1 conferred enhanced tolerance to cold at 4 °C or 2 °C temperatures. Yeast two-hybrid, transient assay, split Luciferase complementation and BiFC assays revealed that PuHHP1 protein can interact with PuICE1. In addition, higher levels of *CBF* and DREB3 transcripts were detected in the transgenic lines, concomitant with the PuHHP1 protein are physically associated with PuICE1 and stimulate PuDREBa transcriptional activities. All of these results demonstrate that *PuICE1* functions positively in cold tolerance by regulating levels of *PuDREBa* transcripts by interacting with PuHHP1. The current study provides new knowledge of the function and underlying mechanism of *ICE1* and expands our understanding of the complex cold signalling network.

## Materials and Methods

### Plant materials and stress treatments

*Pyrus ussuriensis* seedlings were grown at National Center of Pear Breeding, Nanjing Agricultural University, 30 uniform and healthy seedlings were collected from 3-month-old *Pyrus ussuriensis* seedlings and subjected to every stress treatment (dehydration, salt, and cold), about 90–100 leaves were used for every stress treatment. In order to remove physiological and environment influences, shoots of similar length and age of seedling were choose. The shoots were first incubated in distilled water for 48 h at room temperature before being treated with various abiotic stresses, including dehydration, cold, and salt. Stress treatments were performed as follows, for low temperature treatment, the shoots were transferred to 4 °C growth chambers for continuous treatment for 0, 5, 24, 48 and 72 h. For dehydration, the shoots were put in empty flasks, the leaves were collected at 0, 0.5, 1, 3 and 6 h after treatment. For salt treatment, the shoots were dipped into solutions of 200 mM NaCl, 0, 5, 24, 48 and 72 h for salt. The collected samples were then frozen in liquid nitrogen and stored at −80 °C until use for further analysis.

### Isolation and analysis the *PuICE1* gene

The sequence of *AtICE1* (At3g26744) was used to as a bait for a homology search against the Pear Genome database (http://peargenome.njau.edu.cn/), was carried out in order to assemble an *ICE1* conting. To validate the sequence accuracy, RT-PCR was carried out with a primer (GSP1, Table S1) designed according to the cDNA conting. Total RNA was isolated from *Pyrus ussuriensis* seedlings treated at 4 °C for 1.5 h, total RNA was extracted from the treated leaves samples using TRIZOL reagent (TaKaRa, Dalian, China), according to the manufacturer’s instructions. After DNase I treatment, 1 μg of total RNA was used to synthesise first-strand cDNA by the RevertAid^TM^ First Strand cDNA Synthesis Kit (TOYOBO, Japan). The RT-PCR reaction, in a total volume of 50 μl, consisting of 250 ng of cDNA, 1×TransStart FastPfu buffer, 0.25 mM dNTP, 2.5 U of TransStart FastPfu DNA polymerase (TRANS) and 0.5 μM of each primer. The PCR programme consisted of 2 min incubation at 95 °C, followed by 40 cycles of 20 s at 95 °C, 20 s at 55 °C, 60 s at 72 °C, and a 10-min extension at 72 °C. The PCR product was recovered and sub-cloned into pMD18-T vector (TakaRa) and sequenced (UnitedGene, Shanghai, China). Sequence analysis was done in NCBI (http://www.ncbi.nlm.nih.gov/). The multiple aligments was used by ClustalW, and the phylogenetic tree was constructed by the NJ (Neighbor–Joining) method using MEGA 4.0, molecular weight and theoretical isoelectric point (*pI*) were predicted by ExPASy (http://www.expasy.org/tools). Prediction of helix-loop-helix proteins (ICE1) domain was performed on Motif scan (http://myhits.isb-sib.ch/cgi-bin/motif_scan).

### Expression profile of *PuICE1* in different stresses

In order to evaluate transcription level of *PuICE1* under different treatment, qRT-PCR by the SYBR Green dye method was performed according to Huang *et al.*^58^. qRT-PCR reaction was performed in an ABI 7500 Real Time System (PE Applied Biosystems, Foster City, CA, USA). In total volume 10 μl PCR reaction volume, containing 5 μl 2×SYBR Green Real MasterMix (SYBR Green, Applied Biosystems), 50 ng cDNA, 0.25 μM of each primers for *PuICE1* (GSP2, Table S1) or *Tubulin* primer as a control (*Tubulin*, Table S1). Each sample was amplified in four replicates. The reaction program is consisted of 50 °C for 2 min, 95 °C for 10 min, followed by 40 cycles of 95 °C for 15 s, and 60 °C for 1 min.

### Subcellular localization of PuICE1 and PuHHP1

The whole ORF of the *PuICE1* or *PuHHP1* gene was amplified by RT–PCR using primers (GSP3 and GSP4, respectively, Table S1) containing either *NcoI* or *SpeI* restriction site. The PCR products were digested by *NcoI* and *SpeI* and ligated to the 5′ –terminus of GFP in the binary vector pCAMBIA1302 under the control of CaMV 35S promoter to form a fusion construct 35S–PuICE1–GFP or 35S–PuHHP1–GFP. After identified the sequence, the fusion vector and the control vector (pCAMBIA 1302 alone, 35S–GFP) were transferred into *Agrobacterium tumefaciens* strain GV3101 by heat shock. The abaxial surfaces of tobacco leaves were agroinfiltrated with the bacterial suspension (OD600 = 0.5) and then kept in an incubator for 2–3 d, followed by live cell imaging under an inverted fluorescence fluorescence microscope (Olympus BX61, Tokyo, Japan).

### PuICE1 activates transcription and binds to MYC element in the *PuDREBa* promoter

For the transactivation assay, intact or deleted (I1–I5) PuICE1 ORFs were amplified by PCR using primers containing either *BamH*I or *Xho*I restriction sites (GSPF1–GSPF5, Table S1), and the amplicon were inserted into the same enzyme sites of pENTR3C (Invitrogen). The recombinant vectors (pENTR3C–PuICE1) were then fused in frame downstream of the yeast GAL4 DNA–binding domain in pDEST32 by recombination reactions (Invitrogen). The fusion vector and the negative control (pDEST32) were expressed in yeast strain MaV203 (Invitrogen) according to the manufacturer’s instructions. The transformed yeast strains were placed on SD/–Leu/–Trp or SD/–Leu/–Trp/–His medium increasing with or without different concentration of 3–AT (0 and 15 mM) and cultured for 3–4 d to test the expression of the reporter gene *HIS3*. The colony-lift filter assay using 5-bromo-4-chloro-3- indolyl-ß-D-galactopyranoside was carried out based on the instruction manual (Invitrogen) to examine expression of the reporter gene LacZ.

To investigate whether or not PuICE1 can bind to MYC recognition sites in the *PuREBa* promoter, yeast one–hybrid assay was performed as described by the manufacture (Clontech). The ORF of PuICE1 mentioned above was fused to the GAL4 activation domain in the vector pGADT7 digested with *BamH*I and *Nco*I to create pGADT7–*PuICE1* (GSP5, Table S1). A 66–bp oligonucleotide sequence containing triple tandem repeat of a sequence containing MYC (ACTAAGACACATGTGCAATA) was inserted into the pHIS2 vector, generating a recombinant construct of pHIS2–MYC. Thereafter, Both pGADT7–PuICE1 and pHIS2–MYC were co–transformed into yeast strain Y187 to verify the DNA–protein interactions. The transformed cell was placed on SD/–His/–Leu/–Trp medium with or without different concentration 3–AT for 3 d.

### Plant transformation

The full-length coding region of *PuICE1* was sub–cloned to PMD18–T vector using primer containing either *Bgl*II or *BstE*II restriction site (GSP6, Table S1) to get a recombination vector PMD18–T–*PuICE1*. After confirmation by sequencing, the recombination vector was digested by *Bgl*II or *BstE*II, and the target product was inserted into *Bgl*II/*BstE*II linearized binary vector pCAMBIA1301 under the control of 35S promoter. The recombinant vectors were introduced into *A. tumefaciens* strain GV3101 by heat shock after verification by sequencing. The overexpression vectors was used to transform tomato (Mcro–tom). To produce transgenic tomato plants, Agrobacterium-mediated transformation of leaf discs was carried out according to a leaf disc method^50^. Transgenic plants were verified by PCR using a pair of primer CaMV 35S–*PuICE1* (GSP7, Table S1). The *Actin* were used as internal control for tomato.

### Assessment of cold tolerance in the transgenic lines

Thirty-day-old seedings were planted in plastic pots filled with 1:1 mixture vermiculite and soil under a photoperiod of 16 h of light 8 h dark at 25 °C. In order to evaluate the cold tolerance, some of the seedlings were kept at 4 °C for 4 d or 2 °C for 3 d, and the moved to ambient enviroment for further growth. Survival rate was scored after 5 d recovery growth; photos were taken before and after the cold treatment and after the recovery. In additoion, the plants were exposed to cold treatment at chilling temperature (2 °C) for 3 d. The leaves were collected for analysis of Electrolyte leakage, MDA content, chlorophyll content, proline content, POD, SOD, CAT activity were measured after the chilling treatment was stopped, while survival rate was assayed after the recovery at 25 °C for 5 d. All the experiments were repeated three times and the representative results were shown.

### Yeast two–hybrid (Y2H) interaction assays

To confirm an interaction between PuCE1 and PuHHP1, the N–terminal (1–108 aa) of the PuHHP1 was amplified using primer pairs (GSP8, Table S1) and cloned into the *Xho*I and *Kpn*I sites of pDEST22 vector to get AD–PuHHP1, while the truncated PuICE1 was inserted into *Xho*I and *Kpn*I sites of pDEST32 vector to generate BD–PuICE1. truncated PuCE1 (deletion of the transactivation region at aa 46–97) were amplified with primer pairs GSPF4, and inserted into pENTRTM3C (Invitrogen). Fusion proteins were expressed in yeast cells MaV203 and then placed on selection medium ( SD/–Leu/–Trp/–His) supplemented with 5 mM 3–AT at 30 °C for 3 d.

### Plasmid constructs for protoplast transient assays

For transient expression assays using Arabidopsis protoplasts, The *PuDREBa* promoter region was amplified using primers containing *Pst*I and *Nco*I restriction sites (GSP9, Table S1) and cloned by replacing the RD29A promoter in the RD29A–LUC protoplast expression vector. The RD29A–LUC, UQ10–GUS, HBT95–ABI1 protoplast expression plasmid vectors were provided by Zhao *et al. *59. Then, the PuICE1 and PuHHP1 overexpression constructs were used as the effector and inserted into HBT95–ABI1 vector using primers containing *BamH*I or *Pst*I restriction sites GSP10, GSP11, respectively. All the plasmids were confirmed by sequencing. Assays for transient expression in protoplasts were performed as described^51^. All the plasmids used in this assay were extrated with QIA–GEN plasmid Midi Kit. *PuDREBa*:: LUC (5 μg of plasmid per transfection ) was used as reporter. UQ10-GUS (2 μg for per transfection ). PuICE1 and PuHHP1 were used at 3 μg per transfection, respectively.

### Split luciferase complementation assays

For split luciferase complementation assays, the coding sequences of PuICE1 (GSP12, Table S1) and PuHHP1 (GSP13, Table S1) were cloned into pCAMBI–nLUC and pCAMBI-cLUC vectors^60^. *Agrobacterium tumefaciens* GV3101 carrying different constructs was cultured overnight at 28 °C and centrifuged at 4,000 *g* for 10 min. Then the pellet was resuspended to an OD of 1.5 in injection buffer (10 mM MES, pH 5.6, 10 mM MgCl_2_, and 100 μM acetosyringone). Equal amounts of culture were mixed in different combination and kept at room temperature for 3 h. The mixture was then infiltrated into *Nicotiana benthamiana* leaves. Two days after infiltration, luciferase activity was detected with a luminescence imaging system (Princeton Instrument).

### Bimolecular fluorescence complementation (BiFC) assays

For bimolecular fluorescence complementation (BiFC) analysis^61^, the PuICE1 ORF without a stop codon was PCR amplified with primer pair (GSP14, Table S1) and then subcloned into pSPYNE–35S containing the N–terminal fragment of yellow fluorescent protein (nYFP) to get PuICE1–nYFP. Meanwhile, full–length and N–terminal (1–108 aa) of PuHHP1 without a stop codon were amplified using primer pairs (GSP15 and GSP16, respectively) and then inserted into pSPYCE–35S containing the C–terminal fragment of YFP (cYFP) to generate PuHHP1–cYFP or nPuHHP1–cYFP. In the BiFC experiment, PuICE1–nYFP plus PuHHP1–cYFP, PuICE1–nYFP plus nPuHHP1–cYFP were co–tranformed into Arabidopsis mesophyll protoplasts and the transformed protoplasts incubated at 25 °C for 12–20 h. YFP fluorescence in the epidermis was monitored via a universal fluorescence microscope.

### Statistical analysis

The data were statistically processed using the SAS software package (SAS Institute); statistic difference was compared using one–way analysis of variance based on a *t*-test, at the significance levels of *P* < 0.05, *P* < 0.01, and *P* < 0.001.

## Additional Information

**How to cite this article**: Huang, X. *et al.* *ICE1* of *Pyrus ussuriensis* functions in cold tolerance by enhancing *PuDREBa* transcriptional levels through interacting with PuHHP1. *Sci. Rep.* **5**, 17620; doi: 10.1038/srep17620 (2015).

## Supplementary Material

Supplementary Information
