# Supplementary material for: ICE1 of Pyrus ussuriensis functions in cold tolerance by enhancing PuDREBa transcriptional levels through interacting with PuHHP1
Source: Sci Rep. 2015 Dec 2;5:17620. doi: 10.1038/srep17620 (PMC4667267; doi:10.1038/srep17620)

***ICE1* of *Pyrus ussuriensis* functions in cold tolerance by enhancing *PuDREBa* transcriptional levels through interacting with PuHHP1**

Xiaosan Huang^1^, Kongqing Li^2^, Cong Jin^1^, and Shaoling Zhang^1^*

^1^ College of Horticulture, State Key Laboratory of Crop Genetics and Germplasm Enhancement, Nanjing Agricultural University, Nanjing, China, 210095

^2^ College of Rural Development, Nanjing Agricultural University, Nanjing, China, 210095

*Corresponding author: Shaoling Zhang,

Email: slzhang@njau.edu.cn

Xiaosan Huang

Email: huangxs@njau.edu.cn

Kongqing Li

Email:likq@njau.edu.cn

Cong Jin

Email:2012204008@njau.edu.cn

**Supplementary data**

**Table S1.** Primers used in this study.

| **Genes** | **Primers** | **Sequences (**5’-3’**)** | |
| --- | --- | --- | --- |
|  |  | **Forward** | **Reverse** |
| *PuICE1* | GSP1 | **GTGAAATATTACCTGTAAATTTGCAATT** | GGTGAGGATTCTAGGGATCCGTGAATGG |
| *PuICE1*  *Tubulin* | GSP2  *Tubulin* | GAGGCTGAACGGTGGTGTTTGG  TGGGCTTTGCTCCTCTTAC | ctgacatctgaatggccctggatgc  CCTTCGTGCTCATCTTACC |
| *GFP* | GSP3  GSP4 | CCATGGATGCTGCCGAGGCTGAACGGTGGTG (*Nco*I site is underlined)  CCATGGATGAGTCCCAAGGAAGGAGG AGCTC (*Nco*I site is underlined) | ACTAGTCATCATGCCATGGAACCCGATCG (*Spe*I site is underlined)  ACTAGTATTACTGTCACATCCAAAGCTGTTT  (*Spe*I site is underlined) |
| *PuICE1* | GSPF1 | GGATCCATGCTGCCGAGGCTGAACGGTGGTG (*BamH*I site is underlined) | GAATTCCATCATGCCATGGAACCCGATCG (*EcoR*I site is underlined) |
| *PuICE1* | GSPF2 | GGATCCACTAGAACCTCCGCCGCCGCCGCCGG (*BamH*I site is underlined) | GAATTCCATCATGCCATGGAACCCGATCG (*EcoR*I site is underlined) |
| *PuICE1* | GSPF3 | GGATCCTTCAAGTCGATGCTGGAGGTTGAAG (*BamH*I site is underlined) | GAATTCCATCATGCCATGGAACCCGATCG (*EcoR*I site is underlined) |
| *PuICE1* | GSPF4 | GGATCCTCGCCGTCGTCGTCGGTTTTCAAC (*BamH*I site is underlined) | GAATTCCATCATGCCATGGAACCCGATCG (*EcoR*I site is underlined) |
| *PuICE1* | GSPF5 | GGATCCGCTTCGGGTGGCGGTTCGAGTATG (*BamH*I site is underlined) | GAATTCCATCATGCCATGGAACCCGATCG (*EcoR*I site is underlined) |
| *PuICE1*  *PuICE1* | GSP5  GSP6 | GGATCCATGCTGCCGAGGCTGAACGGTGGTG (*BamH*I site is underlined)  AGATCTATGCTGCCGAGGCTGAACGGTGGTG (*Bgl*II site is underlined) | CCATGGCATCATGCCATGGAACCCGATCG (*Nco*I site is underlined)  GGTGACCCTACATCATGCCATGGAACCCGATCG (*BstE*II site is underlined) |
| ***35S-****PuICE1* | GSP7 | ACTATCCTTCGCAAGACCCT | CATCATGCCATGGAACCCGATCG |
| *Actin*  *PuHHP1*  *PuDREBa*  *PuICE1*  *PuHHP1*  *PuICE*  *PuHHP1*  BiFC | *Actin*  GSP8  GSP9  GSP10  GSP11  GSP12  GSP13  GSP14  GSP15  GSP16 | TGGGCTTTGCTCCTCTTAC  CTCGAGATGAGTCCCAAGGAAGGAGGAGCTC (*Xho*I site is underlined)  CTGCAGACCGGACCAGATGGTATTGTCCTTGG (*Pst*I site is underlined)  GGATCCTATGCTGCCGAGGCTGAACGGTGGTG (*BamH*I site is underlined)  GGATCCTATGAGTCCCAAGGAAGGAGGAGCTC (*BamH*I site is underlined)  CACCATGCTGCCGAGGCTGAACGGT  CACCATGAGTCCCAAGGAAGGAGG  GGATCCATGCTGCCGAGGCTGAACGGT (*BamH*I site is underlined)  ACTAGTATGAGTCCCAAGGAAGGAGG AGCTC (*Spe*I site is underlined)  ACTAGTATGAGTCCCAAGGAAGGAGG AGCTC (*Spe*I site is underlined) | CCTTCGTGCTCATCTTACC  GGTACCGGTTTCGTTATGCCACCGGAACACGC (*Kpn*I site is underlined)  CCATGGGGCTGGGCAGGCATGGAATCCACG (*Nco*I site is underlined)  CTGCAGCATCATGCCATGGAACCCGATCG (*Pst*I site is underlined)  CTGCAGTTAATTACTGTCACATCCAAAGCTG (*Pst*I site is underlined)  CATCATGCCATGGAACCCGATCG  ATTACTGTCACATCCAAAGCTGTTT  ATCGATCATCATGCCATGGAACCCGATCG (*Cla*I site is underlined)  GGTACCATTACTGTCACATCCAAAGCTGTTT(*Kpn*I site is underlined)  GGTACCGGTTTCGTTATGCCACCGGAACACGC (*Kpn*I site is underlined) |
| AK319984 | APX | CACTTTCTGGGGCACACACT | CAGCATCTGTAGGCAAAACT |
| AK319476 | SOD | GCCCACTCAATCTTCACCAC | AGTGACAACCCCCTCAACAT |
| AK320529 | CAT | TCTTCTACGATGATGTTTGTCTCC | GGATAGTTTCCAGCAGCAAT |
| AK323053 | ADC | GTGATGGTGGGCGGTATTAT | caacggcagctttcatgtta |
| AK328401 | CBF1 | CTGCCTATCCCTGCTTCCTC | CCTCCTCATCCACGAAGTCA |
| AK322452 | DREB3 | ATGGAATGGAGCAACAGGGT | CGACCCAGATTGCTTCATTG |

**Fig. S1**. Generation and molecular identification of transgenic tomato plants overexpressing *PuICE1.*


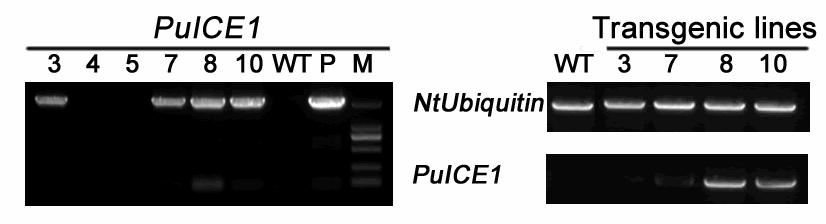

Supplement: Supplementary Information [file srep17620-s1.doc]
